# The Arabic EAT-10 and FEES in dysphagia screening among cancer patients: a comparative prospective study

**DOI:** 10.1038/s41598-024-58572-z

**Published:** 2024-04-22

**Authors:** Da’ad Abdel-Hay, Osama Abdelhay, Hamza A. Ghatasheh, Sameer Al-Jarrah, Suhaib Eid, Mutaz A. Al Tamimi, Ibrahim Al-Mayata

**Affiliations:** 1 Department of Surgery, King Hussein Cancer Centre, Queen Rania Street, Amman, 11941 Jordan; 2 Department of Data Science and Artificial Intelligence, Princess Sumaya University of Technology, Khalil Saket Street, Amman, 1438 Jordan; 3 Department of Radiation Oncology, King Hussein Cancer Centre, Queen Rania Street, Amman, 11941 Jordan; 4 Royal Rehabilitation Center, King Hussein Medical Center, King Abdullah II St 230, Amman, Jordan; 5 Department of Nursing, King Hussein Cancer Centre, Queen Rania Street, Amman, 11941 Jordan

**Keywords:** Cancer, Health care, Oncology

## Abstract

Head and neck cancer treatments, such as radiotherapy, chemotherapy, and surgery, have diverse effects on patients, leading to dysphagia as a significant post-treatment issue. This study aims to evaluate the effectiveness of the Arabic version of the EAT-10 screening instrument (A-EAT-10) using Fiber-Optic Endoscopic Evaluation of Swallowing (FEES) as an imperfect gold standard. Additionally, we seek to establish a correlation between A-EAT-10 and PEG tube insertion in head and neck cancer (HNC) patients. Our sample comprised 130 head and neck cancer patients with varying cancer types at King Hussein Cancer Center (KHCC). We followed these patients throughout their distinct treatment plans up to one month after their final treatment session. During follow-up visits, we administered the A-Eat-10 instrument to monitor dysphagia. FEES were conducted at the initial and concluding visits to compare results with A-EAT-10 scores. The results in our tests, assuming independence or dependence, demonstrated excellent agreement. A-EAT-10 exhibited outstanding predictive capabilities with an AUC ranging from 93 to 97%. A-EAT-10 tended to slightly overestimate dysphagia at later treatment stages by approximately 20% compared to FEES, with an RR of 1.2 (95% CI 0.91, 1.56, *p*-value = 0.21), indicating statistical insignificance. In conclusion, A-EAT-10 is an excellent option for dysphagia evaluation, offering non-invasive, straightforward, and cost-effective advantages compared to FEES. Its utility extends to predicting the need for PEG tube insertion at initial patient visits, making it a valuable tool for informed treatment decisions. Notably, A-EAT-10 demonstrates a diminishing correlation with FEES over time.

## Introduction

Head and neck squamous cell carcinoma (HNC) stands as one of the most prevalent cancers among the Jordanian population, affecting approximately 11% of men and 2.5% of women^1^. The primary treatment modalities for HNC, encompassing surgery, radiotherapy, and chemotherapy, often result in enduring physical, social, and psychological challenges due to debilitating side effects^2^. Despite the evolution of treatment strategies, a prominent complication arising from all forms of treatment contributing to increased morbidity and mortality in HNC patients is oropharyngeal dysphagia (OD)^3^. A comprehensive assessment approach is imperative to identify individuals with OD, facilitating the prevention and management of adverse outcomes^3^. A critical diagnostic tool speech-language pathologists (SLPs) employ is Fiber-Optic Endoscopic Evaluation of Swallowing (FEES), considered the ‘Gold Standard’ for diagnosing swallowing disorders and assessing OD, including determining aspiration^4^. FEES emerges as a safe and reliable tool for diagnosing and managing dysphagia^5,6^. The Arabic Eating screening tool (A-EAT-10), a self-administered survey validated in 2016, is a self-report screening tool for OD^7^. This tool allows patients to assess their swallowing subjectively and provides clinically relevant information to monitor treatment response in various HNC types^7^.

Despite the widespread use of the general EAT-10 screening tool in SLP clinics, there is a need to tailor this scale for highly prevalent populations with OD^3^. Notably, the specific A-EAT-10 exhibits the poorest correlation studies between A-EAT-10 and other dysphagia assessment tools such as the Penetration-Aspiration scale (PAS), an 8-point scale characterizing the depth and response to airway invasion during FEES. The PAS is a scale ranging from 1 (materials do not enter the airway to 8 ( materials enter the airway, pass below the vocal folds, and no effort is made to eject)^8^. While several studies affirm the reliability of PAS and its confidence level ranging between 70 and 80%, variations in results between the general EAT-10 and PAS correlation, have been moderately observed, especially in determining OD severity among HNC patients^3,9^.

A nuanced understanding of the relationship between A-EAT-10 and swallowing physiology, incorporating instrumented assessments like FEES with the interpretation of PAS, could enhance clinical care. Such improvements may lead to a reduction in the severity of OD. Percutaneous endoscopic gastrostomy (PEG), a widely utilized procedure locally and internationally, enhances the nutritional status among HNC patients^10,11^. PEG tubes positively impact feeding and nutrition, yet prolonged usage can pose risks, ranging from minor complications like inflammation to major issues such as severe aspiration pneumonia^12,13^. Despite one study emphasizing the significance of predicting the need for PEG tube insertion before treatment^14^, no definitive global or local studies establish the ideal duration for retaining PEG tubes, especially in patients diagnosed with early cancer stages.

This study advances our understanding of the commonly used Arabic screening tool (A-EAT-10) for HNC populations with OD. The study’s primary objective is to determine a robust correlation between A-EAT-10 and FEES results among HNC patients, using PAS for FEES interpretation. The secondary objective involves monitoring the necessity of PEG tube insertion, particularly in cases where A-EAT-10 scores fall within normal limits. Still, dysphagia is detected during FEES, necessitating precautionary PEG placement before treatment initiation. If inserted, these findings may significantly contribute to clinical decision-making, offering insights into the appropriate timing for PEG tube implementation and removal.

## Material and methods

The present prospective cohort study was conducted following the Declaration of Helsinki^15^. The KHCC Institution review board approved the study with IRB number 19 KHCC 127.

### Participants

Participants underwent a thorough swallowing and speech assessment and were diagnosed with any HNC according to the criteria proposed by King Hussein Center guidelines^16^. All HNC patients who underwent a FEES examination at the speech and swallowing outpatient Clinic between 2020 and 2022 were included in the study. This study’s total clinical data set consisted of 130 patients, with 108 males and 22 females. These patients were selected from those seen at a routine speech outpatient clinic. They were invited to participate if they met the following inclusion criteria: adults, newly diagnosed with any HNC (including laryngeal cancer, nasopharyngeal cancer, oropharyngeal cancer, thyroid cancer, multilocation as Neck Cancer, Tongue and Maxilla Cancer), having cancer at any stage (T1, T2, T3, or T4), Most of all tumours arises from HNC mostly become as squamous cell carcinoma or one of its variants. Treatment approaches are contingent upon tumour staging. For instance, in the case of laryngeal cancer or nasopharyngeal cancer, radiotherapy is typically recommended for T1(a,b) N0 stages. Surgical intervention becomes the preferred course for resectable tumours in any T stage with N1-N3, M0 conditions. However, for tumours considered non-resectable, concurrent chemoradiotherapy is the preferred treatment modality. This nuanced approach underscores the importance of tailoring treatment strategies based on the specific characteristics and staging of the malignancy^10,16^.

Exclusion criteria included a medical history of gastroenterological, respiratory, rheumatologic, metabolic, or hematologic disorders and any previous completion of radiotherapy, chemoradiotherapy, or multimodality treatments. Patients who had undergone surgery, such as total laryngectomy, tracheostomy, or PEG tube insertion, were also excluded from the study.

Informed consent was obtained from all patients during regular outpatient speech and swallow clinic visits.

### Swallowing protocol

In the dysphagia outpatient clinic, a standardized examination protocol was implemented for regular healthcare assessment. The protocol encompassed a clinical ear, nose, and throat examination conducted by a laryngologist to assess the integrity of cranial nerves. The A-EAT-10 is a valid tool suitable for screening dysphagia-related problems in the Arabic-speaking population. Internal consistency and test–retest reliability were evaluated, and the author studied content and clinical validity^7^.

The A-EAT-10 is a self-administered, symptom-specific outcome instrument for dysphagia. It comprises ten statements patients rate on a scale of 0 to 4, with 0 indicating no problem and 4 signifying a severe problem^7^. During their regular visits to the speech clinic, any patients with or without dysphagia who met the inclusion criteria were asked to complete the A-EAT-10 questionnaire by themselves. For patients who required assistance due to literacy issues, a SLP facilitated the process by verbally presenting the questions and recording their responses. The A-EAT-10 offers an overall screening of dysphagia and its associated symptoms^7^. A senior SLP and ENT physician performed the standardized fibre optic endoscopic evaluation of swallowing (FEES) examination. An OtoPront PES PILOT HDpro stroboscope flexible endoscope with a 2.7 mm diameter, equipped with Video Nasopharyngoscopes VN-S and VN-P (CHIP-ON-THE-TIP) and an automatic switching system from Berlin, Germany, was utilized. All recorded videos were processed using the PILOT system and stored in an anonymous format^17^. Each FEES examination adhered to the clinical practice guidelines for fibre optic examination in the adult population^18^. Patients were comfortably seated in a chair reclined between 75 and 90 degrees, with their arms resting on the armrests and their heads in a neutral position, ensuring the best posture for the examination. Local anaesthetic drugs, such as lidocaine spray, were not used to avoid altering pharyngolaryngeal sensibility^19^. The endoscope was introduced into the most expansive nasal cavity and maintained just below the uvula to maximize the field of view, including the larynx, the glossoepiglottic valleculae, and the pyriform sinuses^19^. During the FEES examination, three different food textures were administered to evaluate swallowing safety and efficiency:

- **Liquid:** Thin liquid at room temperature (IDDSI Level 0) was used for thin liquid trials^20^.
- **Moderate thick liquid:** Room temperature yoghurt (IDDSI Level 4) was employed for moderate thick liquid trials^20,21^.
- **Solid:** A quarter and half of an 8-g piece of dry bread (4 g per trial; IDDSI Level 7) were used for solid trials^20^.

Some food bolus consistencies were not administered to all patients for safety reasons and to minimize the risk of severe aspiration. Consequently, the study included only subjects who had undergone at least one trial with thin or moderate thick liquid consistencies.

FEES examinations were rated independently by three operators using the video files. Two of them were speech and language therapists (SLPs and one Senior ENT physician.), all three with at least five years of experience in FEES examinations. SLPs and ENTs were blind to each other and participants’ data, since videos were stored anonymously. Two independent SLPs rated the videos using validated ordinal scales for swallowing safety and efficiency; inter-rater reliability between the two raters was analyzed. In case a difference > 1 level at each FEES rating scale occurred between the two raters, a 3rd SLP assessed the videos and decided on both ratings, The third SLP acted as a conflict resolution party^22^.

The table in the supplemantray file (Table S1) illustrates the data collection of A-EAT-10 and the timing of the FEES application. All patients attended regular follow-up visits throughout treatment, and each received ongoing support from an SLP. The SLP’s role includes maintaining, educating, and offering counselling concerning dysphagia symptoms, providing guidance on PEG tube utilization (if applicable), and administering swallowing exercises.

### Statistical analysis

The sample size is calculated based on Cohen’s kappa coefficient for the agreement between two raters^23^. The sample size calculation assumed a minimum acceptable kappa (${\kappa }_{0}$) of 0.61 (moderate to substantial agreement), and an alternative expected kappa (${\kappa }_{1}$) to be 0.4 (fair agreement). The expected prevalence of dysphagia is 30% among the participants. The power of the test is 80%, with a type I error equal to 0.05. These assumptions resulted in an estimated required sample size of 132 patients. At the beginning of the study, 143 participants were recruited. Only 130 who completed the study. The reasons for dropouts and attrition included ten patients who had undergone tracheostomy and PEG tube insertion before the start of treatment, following KHCC guidelines. Additionally, two patients unfortunately passed away during the follow-up period, and one was referred to palliative care.

The analysis’s general theme is measuring agreement between diagnostic tools under the imperfection of the gold standard and latent class analysis. The methods in this area are somewhat confusing because of many issues related to the theory. The number of alternatives and lack of consistency in the literature are significant sources of confusion even for statisticians^22,24–26^. We developed a simple protocol to identify the ‘best’ method(s) we can employ for the dataset in hand, considering the nature of the data^22,24–26^.

The first consideration is to identify the aim of this comparison, as mentioned earlier, to validate the A-EAT-10 (self-report-based questionnaire) Arabic version using a traditional diagnostic tool as a benchmark, i.e., the FEES test. The established traditional method does not always reflect the truth, and there is some inaccuracy (error). Hence, considering FEES a ‘gold standard,’ i.e., not to have measurement errors, will be inappropriate because even this method does not produce accurate results^27^.

The second consideration is the dependency issue. Since the two tests are performed on the same patient, there is a risk of dependency. We will discuss several methods in the protocol developed under the independence assumption^28,29^.

Given these considerations, we considered using measures of the agreement. Additionally, we used sensitivity and specificity to assess the validity of A-EAT-10 given the imperfection of the gold standard, i.e., the FEES test and the data dependency. The protocol for analyzing the agreement is attached as a supplementary file.

Intra-class correlation test (ICC) with a single random effect^30^. The advantage here over the kappa coefficient is the different randomness parts introduced in the model. As in our case, the single random effect (mixed effect) assumes the patients’ randomness only, and the two assessment methods are fixed^31^. The interpretation of ICC is like kappa. (See Table S2 in supplemantary file for the criteria).

The traditional sensitivity–specificity analysis will include the receiver operator curve (ROC) and the area under the curve (AUC). However, this approach will be adjusted to account for the imperfection of the gold standard^32^. Corrections with prevalence rates between 0.1 and 0.9 show similar results in detecting reality, with superior results under conditional independence between the tests for Staquet et al. correction^28^. The correction for the imperfection required the establishment of the imperfect gold standard sensitivity and specificity from previous studies or a test group^28,29,33,34^. Previous studies show that FEES has approximately 80–87% sensitivity and 81–100% specificity in detecting dysphagia^28,29,31–44^. We are correcting based on these levels. There is no established sensitivity–specificity regarding our search for HNC patients. Therefore, we will use the studies’ lowest and highest reported levels as our thresholds for the imperfect gold standard: sensitivity (80% and 87%) and specificity (81% and 100%). Alternating between different levels of sensitivity and specificity will act as a sensitivity analysis. This process will provide an upper and lower bound for the specifications of the EAT-10 test.

Random Effects Latent Class Analysis (RE-LCA) is employed under the assumption of conditional dependence^45^. This approach assumes two latent classes (with and without the condition): dysphagia versus normal. The condition’s actual status (dysphagia vs. normal) is unknown, and the best reference test, FEES, indicates the status. This test is referred to as an imperfect gold standard. Therefore, the actual status of the condition is ‘latent’. Like the previous point, the sensitivity and specificity of FEES are assumed to be known. All analyses were performed using R Statistical Software (v4.2.1; R Core Team 2022).

### Ethical approval

Permission to use the Arabic-EAT-10 for the study was obtained from the Copyright holder, Dr.Tamer Mesallam. An ethical approval for study protocol was approved by King Hussein Cancer center ethical committee, approved the study with IRB NO: 19 KHCC 127. A written informed consent was obtained from all patients after explaining the objectives of the study and ensuring the freedom to refuse without affecting in daily clinical practice.

## Results

The patients’ characteristics are described in Table 1. The average age of the patients is 56 years, with the majority being males (82.3%), Jordanians (97%), and smokers (73.1%).

**Table 1:** Patients’ characteristics (n = 130).

Characteristic	Mean ± SD*	Number (%)
Age	56 ± 12.1	
Gender		
Female		23 (17.7)
Male		107 (82.3)
Total		130 (100)
Height	1.69 ± 0.1	
Weight	74.9 ± 16.9	
BMI**	26.1 ± 5.3	
Nationality		
Jordanian		126 (97)
Non-Jordanian		4 (3)
Total		130 (100)
Smoking status		
Smokers (including ex-smokers of less than one year)		95 (73.1)
Ex-smokers (ex-smokers of more than one year)		14 (10.8)
Non-smoker (Never smoked or ex-smokers of ten years or more)		21 (16.1)
Total		130 (100)
Tumour location		
Larynx		71 (54.6)
Nasopharynx		33 (25.4)
Multilocation		6 (4.6)
Thyroid		5 (3.8)
Maxillary sinus		4 (3.1)
Oral cavity/tongue		4 (3.1)
Oropharyngeal		7 (5.4)
Total		130 (100)
Staging		
T0		3 (2.3)
TI		33 (25.4)
TII		28 (21.5)
TIII		39 (30.0)
TIV		27 (20.8)
Total		130 (100)
Treatment plan		
Chemotherapy		8 (6.2)
Concurrent chemoradiotherapy		43 (33.1)
Palliative chemotherapy		1 (0.8)
Palliative radiotherapy		1 (0.8)
Radiotherapy		42 (32.3)
Surgery		35 (26.9)
Total		130 (100)

*SD, Standard deviation.

**BMI, Body mass index.

**Figure 1:**
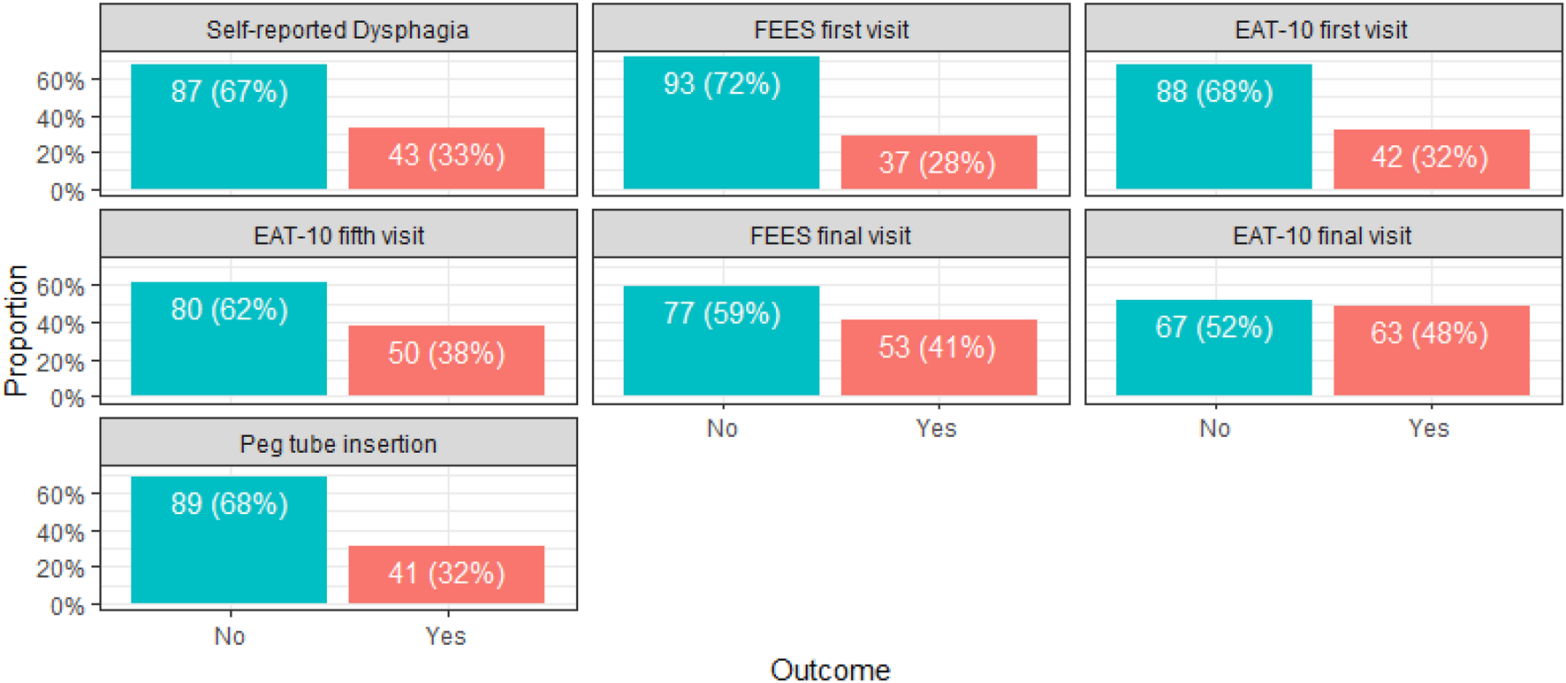
Dysphagia among patients either as self-reported, FEES or A-EAT-10 diagnosis and patients who underwent PEG tube insertion per the treatment protocol (n = 130).

The participating patients reported their swallowing sensation on their first clinic visit (self-reporting dysphagia). Also, they underwent two tests (FEES and A-EAT-10). Both tests were repeated at the final visit (one month after finishing treatment). At the fifth visit, the patients received A-EAT-10 and were diagnosed again with dysphagia without a FEES test. PEG tube insertion was measured per the patients’ treatment protocol. The numbers and percentages for each category are summarized in Table 2 and Fig. 1.

**Table 2:** Dysphagia among patients either as self-reported, FEES or A-EAT-10 diagnosis and patients who underwent PEG tube insertion per the treatment protocol (n = 130).

	Yes—Dysphagia (%)	Normal (%)	Total
Self-reported dysphagia	43 (33%)	87 (67%)	130 (100%)
FEES first visit	37 (28%)	93 (78%)	130 (100%)
A-EAT-10 first visit	42 (32%)	88 (68%)	130 (100%)
A-EAT-10 fifth visit	50 (38%)	80 (62%)	130 (100%)
FEES final visit	53 (41%)	77 (59%)	130 (100%)
A-EAT-10 final visit	67 (48%)	63 (52%)	130 (100%)
PEG tube insertion per protocol	41 (32%)	89 (68%)	130 (100%)

The analytical protocol described in the methods part is explained below. The first step in the protocol is using Cohen’s kappa of measures agreement. The results compared the agreement in diagnosing dysphagia at the first and final visits by the patients. Since the weighted and unweighted results are identical, we only report the result as a kappa value (Table 2). The results suggest approximately perfect agreement between the two methods on the first visit. However, over time and one month of treatment, the 95% confidence interval for kappa of the final visit suggests a good to a near-perfect agreement.

The results of the agreement are reported in Table 3. And the (Table 4) below shows the results for ICC are identical to kappa, with the only exception of narrower confidence intervals. The agreement is almost perfect at the beginning of the treatment cycle and slightly decreasing at the end. The one-way ICC is also employed similarly to the first and final visits of the patients.

**Table 3:** kappa coefficient of agreement test at different visits between FEES test and A-EAT-10 (n = 130).

Visit	Kappa value (κ)	95% confidence interval of kappa (κ)	
		Lower bound	Upper bound
First	0.91	0.83	0.99
Final	0.82	0.72	0.91

**Table 4:** Intra Class Correlation (ICC) agreement at different visits between the FEES test and A-EAT-10 (n = 130).

Visit	ICC	95% Confidence interval of ICC	
		Lower bound	Upper bound
First	0.91	0.88	0.94
Final	0.82	0.75	0.88

### Under independence assumption

**Figure 2:**
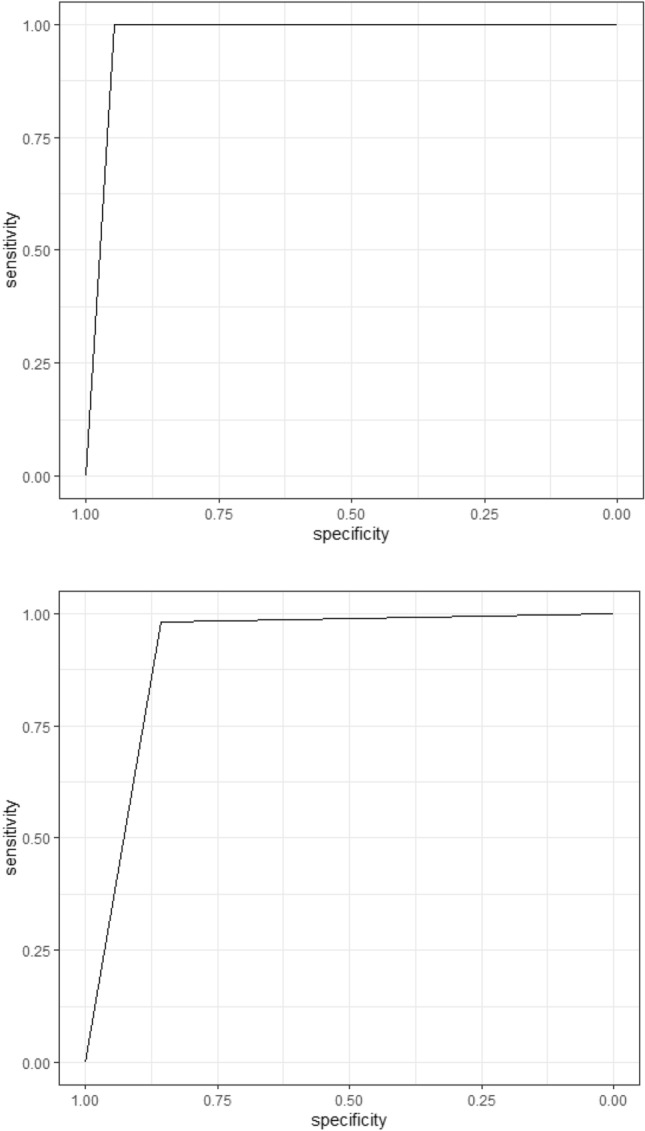
ROC and AUC under the assumption of independence and Staquet correction.

Alternating between the lower and upper sensitivity of the FEES test and using Staquet et al. correction, the sensitivity of the A-EAT-10 is 88–91%, and the specificity is 97–100%. The area under the curve ranged between 92 and 97%. Figure 2 shows the lower and upper bounds of the AUC.

The best results are obtained on the first visit. At the first visit, the minimum sensitivity obtained was 90%, and the maximum was 91%. At the same time, the specificity equalled 100%. The final visit produced the lower bounds.

### Under dependence assumption

Under dependence, we used the RE-LCA model to fit a random model with two classes. This process was employed for the first and final visits. The results in the first visit (FEES v1 vs. A-EAT-10 v1) show, under this model, 93% sensitivity of A-EAT-10 compared to FEES. The specificity is approximately 97%. On the final visit, these numbers decreased. The sensitivity decreased to 87%, and the specificity decreased to 91%. The marginal probability shows that FEES has a 28.5% probability of diagnosing dysphagia at the first visit. A-EAT-10 has a higher probability of 32.3%. In the final visit, FEES has a marginal probability of 40.8% of diagnosing dysphagia, and A-EAT-10 has 48.5%; the Relative Risk (RR) = 1.19, with a confidence interval (0.91, 1.56), and *p*-value = 0.21.

## Discussion

The A-EAT-10 tool is a widely used clinical screening instrument designed to evaluate swallowing difficulties, and it has demonstrated good validity and predictability^7^ The diverse range of treatments available for HNC patients, coupled with the inherent variability in tumour characteristics, contributes to the varying severity of OD. Even among T1 tumours, patients may experience mild symptoms and alterations in swallowing mechanisms, including changes in taste. However, as the tumour progresses to the T3-T4 stages, the severity of OD intensifies. Timely detection of OD is crucial for facilitating prompt initiation of swallowing rehabilitation and nutritional support. Nevertheless, there is a need for clarification on which patients should be routinely monitored for OD. Establishing clear criteria for monitoring is essential to ensure that individuals at risk receive timely interventions to address swallowing difficulties and nutritional challenges associated with their specific stage of HNC.

This work used different protocols under different assumptions to establish boundaries for the sensitivity and specificity of the A-EAT-10 test compared to the standard tests such as FEES. The tests showed that A-EAT-10 approximates the FEES test on the first visit. The different approaches implemented in this work showed that at the first visit, the A-EAT-10 test has a high agreement with positively diagnosing dysphagia and approximately perfect agreement on negatively diagnosing dysphagia compared to FEES. There is a tendency of A-EAT-10 to diagnose dysphagia. This result was consistent across the visits. The RE-LCA model confirmed this result as the probability of diagnosing dysphagia was higher in A-EAT-10 on the first and final visits.

Based on the study’s findings, it’s evident that the A-EAT-10 questionnaire plays a crucial role in the early evaluation of HNCpatients with a different type of staging or tumour. It can assist in determining whether a PEG tube is needed proactively, as a preventive measure in anticipation of potential nutritional support, or reactively, when clinical indications for enteral feeding become apparent. Monitoring the swallowing process during follow-up appointments at the speech and swallowing clinic at KHCC, may assess the timing and necessity of tube usage. Current study found that 41% of patients reported difficulty swallowing during the first visit, confirmed using the FEES or A-EAT-10 questionnaire. Therefore, 41% of these patients had a feeding tube inserted before the treatment started. This result means that patients who reported difficulty swallowing, and whose difficulty was confirmed during the first evaluation by A-EAT-10, had results that were consistent with their PEG tube placement, according to the protocol followed at the KHCC. Moreover, Laryngoscopy is often uncomfortable for some people because it enters through the nose to the throat, and the patient is asked to make sounds or eat something to ensure no aspiration risk^46^. Therefore, the accuracy rate of the A-EAT-10 questionnaire is significant to us in the initial visits for assessment, as it reduces the use of invasive or uncomfortable procedures such as FEES^46,47^.

A simple, swift, and reliable tool like A-EAT-10 in the early stages aids in identifying the requirement for a PEG tube and it has the potential to reduce the necessity for patients without dysphagia, when feeding tube procedures are conducted. This valuable information guides us in making post-treatment decisions regarding the retention or removal of the PEG tube.

Various factors influence the physician’s decision to recommend inserting a feeding tube, including the patient’s anticipated radiation dose, concurrent chemotherapy with radiation, and their ability to tolerate the treatment. Nevertheless, specifying a precise timeframe for PEG tube placement may assist physicians in convincing patients to opt for the tube’s insertion, thereby alleviating the psychological stress patients often experience during their treatment phase^45^. PEG tube duration varied among patients, with a median duration of 168 days observed among HNC) cases^48^. A study found a link between long-term PEG tube dependency and poorer survival^49^. Another prospective cohort study suggested that long-term tube dependence significantly decreased when patients partially used the PEG tube while maintaining continuous oral intake^50^. In addition, dysphagia was prevalent, with 41% of patients reporting it during their initial visit in our study. A similar proportion persisted until the final sessions, where the A-EAT-10 questionnaire revealed that 48% of patients reported dysphagia. Furthermore, 32% of patients required the tube one month after the treatment. It’s important to note that the questionnaire may yield a higher percentage than FEES, as it covers a broader range of swallowing symptoms and details, including questions about appetite and saliva. Moreover, the questionnaire’s agreement with FEES decreased in later stages, especially during the final visit, which included a re-test one-month post-treatment. This decrease could be attributed to patients undergoing additional treatments, such as chemotherapy after radiation, which can exacerbate swallowing difficulties. It may also result from patients completing adjuvant radiation treatment after surgery, heightening the sensation of dysphagia even when FEES results appear normal.

Although the A-EAT-10 did not precisely specify the required duration for retaining the PEG tube after treatment, it is clear that keeping the PEG tube for at least one month post-treatment is essential to maintain good nutritional status among patients. this study has provided support for this, explaining that patients who undergo PEG tube placement after one month can maintain nutritional support.

Despite the challenges posed by data collection during the COVID-19 pandemic, the preliminary findings of the current study suggest that the A-EAT-10 questionnaire holds promise as a valuable assessment tool for predicting dysphagia symptoms and the need for PEG tube insertion in the early stages of treatment. It was observed that keeping a PEG tube for a month after treatment completion can be beneficial, and subsequent re-evaluation helps determine whether PEG tube removal is necessary.

This study is subject to several limitations. Firstly, the sample size of enrolled patients is relatively small, although it is consistent with previous studies. This limitation arises from unforeseen complications associated with the disease and data collection during the COVID-19 pandemic. Ideally, a larger patient cohort would have been preferred to enhance the study’s statistical power. Consequently, the results presented in this study should be interpreted cautiously, especially true given the subjective nature of PASeffects and potential variations in score interpretations among clinicians. In light of this, an alternative gold standard should be considered to guide treatment plans and validate the accuracy of the A-EAT-10 tool.

## Conclusion

In conclusion, there is a discernible decrease in correlation between A-EAT-10 and FEES over time. Nevertheless, its sensitivity and specificity remain acceptable even after a month. Noteworthy is A-EAT-10’s higher sensitivity than specificity and its superior diagnostic probability for dysphagia during initial assessments, presenting it as a valuable prelude to the costly and somewhat discomforting instrumental evaluation of dysphagia, such as FEES. A-EAT-10 can be considered as a beneficial screening tool for identifying dysphagia symptoms in HNC patients and contributing to the timely management of PEG tube insertion at the first visit.

### Supplementary Information

Supplementary Information.

## Data Availability

The datasets generated during and/or analyzed during the current study are available from the corresponding author on reasonable request.

## References

[1] Health-MOH Mo. in *Jordan Cancer of Registry.* (Amman, MOH, 2018).

[2] Caudell JJ, Gillison ML, Maghami E, Spencer S, Pfister DG, Adkins D, Darlow SD (2022). NCCN Guidelines insights: Head and neck cancers, version 1.2022: Featured updates to the NCCN guidelines. J. Natl. Compr. Cancer Netw..

[3] Bartlett, R. S., Kenz, M. K., Wayment, H. A. & Thibeault, S.L. Correlation between EAT‑10 and aspiration risk difers by dysphagia etiology. *Dysphagia *1–10 (2021). 10.1007/s00455-021-10244-0 33486590

[4] Fattori B, Giusti P, Mancini V, Grosso M, Barillari MR, Bastiani L, Nacci A (2016). Comparison between videofluoroscopy, fiberoptic endoscopy and scintigraphy for diagnosis of oro-pharyngeal dysphagia. Acta Otorhinolaryngol. Ital..

[5] Miller CK, Schroeder JW, Langmore S (2020). Fiberoptic endoscopic evaluation of swallowing across the age spectrum. Am. J. Speech-Lang. Pathol..

[6] Pizzorni, N., Berruti, G., Giaccone, M., Zenga, F., Maestri, R., Cossa, M. & Schindler, A. The role of fiberoptic endoscopic evaluation of swallowing in the managment of patiemnts with swallowing problems. *Dysphagia. *607–615 (2019).

[7] Farahat M, Mesallam TA (2015). Validation and cultural adaptation of the Arabic version of the eating assessment tool (EAT-10). Folia Phoniatr. Logop..

[8] Kitila, M., Borders, J. C., Krisciunas, G. P., McNally, E. & Pisegna, J. M. Confidence, accuracy, and reliability of penetration-aspiration scale ratings on flexible endoscopic evaluations of swallowing by speech pathologists. *Dysphagia. *1–10 (2023). 10.1007/s00455-023-10635-5 37980635

[9] Zuniga SAEBJN (2018). Utility of eating assessment tool-10 in predicting aspiration in patients with unilateral vocal fold paralysis. Otolaryngol. Head Neck Surg. (United States).

[10] KHCC KHCC. in *The Guidlines of Nasophryngeal Managment.* (Amman, KHCC, 2021).

[11] Cady, J. Nutritional supporduring radiotherapy for head and neck cancer: the role of prophylactic feeding tube placement. *Clin J Oncol Nurs 11*(16), (2007). 10.1188/07.CJON.875-880 18063546

[12] Hausmann J, Kubesch A, Müller von der Grün J, Goettlich CM, Filmann N, Oliver Tal A, Blumenstein I (2019). Prophylactic percutaneous endoscopic gastrostomy in patients with head and neck cancer: Influence on nutritional status, utilisation rate and complications. Int. J. Clin. Pract..

[13] Murphy, B.A., Deng, J. Advances in supportive care for late effects of head and neck cancer. *Clin. Oncol.* 1861–1871 (2021). 10.1200/JCO.2015.61.3836 26351334

[14] Lee SCWTJ, Chu PY (2019). Predictors of weight loss during and after radiotherapy in patients with head and neck cancer: a longitudinal study. Eur. J. Oncol. Nurs..

[15] Dechaphunkul TSP, Geater SL, Dechaphunkul A (2022). Utility of prophylactic percutaneous endoscopic gastrostomy tube in head and neck cancer patients undergoing concurrent chemoradiation: A prospective observational cohort. Am. J. Otolaryngol..

[16] KHCC KHCC. in *The Guidline of Managment Laryngeal Cancer.* (Amman, KHCC, 2021).

[17] Company, O. Otopront product. In. *Otopront PES PILOT HDpro stroboscope* (2024).

[18] Langmore SE, Scarborough DR, Kelchner LN, Swigert NB, Murray J, Reece S, Rule DK (2022) Tutorial on clinical practice for use of the fiberoptic endoscopic evaluation of swallowing procedure with adult populations. *Am. J. Speech Lang. Pathol*. 187 (2022). 10.1044/2021_AJSLP-20-00348 34818509

[19] Mozzanica, F.,Pizzorni, N., Eplite, A. *et al*. Swallowing characteristics in patients with multiple system atrophy analyzed using FEES examination. *Dysphagia.* (2023). 10.1007/s00455-023-10619-5 PMC11127813 37733099

[20] IDDSI IDDsi. The IDDSI Framework. *International Dysphagia Diet standardisation initiative IDDSI.* 2024. https://iddsi.org/Framework. Accessed 2 28.

[21] IDDSI. IDDSI. *IDDSI.* 2023. Chrome-extension://efaidnbmnnnibpcajpcglclefindmkaj/https://iddsi.org/IDDSI/media/images/Complete_IDDSI_Framework_Final_31July2019.pdf. Accessed 10 16.

[22] Morgan CJ, Aban I (2016). Methods for evaluating the agreement between diagnostic tests. J. Nucl. Cardiol..

[23] Donner A, Eliasziw M (1992). A goodness-of-fit approach to inference procedures for the kappa statistic: Confidence interval construction, significance-testing and sample size estimation. Stat. Med..

[24] Waikar SSBRES, Bonventre JV (2013). Imperfect gold standards for biomarker evaluation. Clin. Trials (London, England).

[25] Guthrie D, Kraemer HC (1992). Evaluating medical tests: Objective and quantitative guidelines. J. Am. Stat. Assoc..

[26] Pepe, M. *The Statistical Evaluation of Medical Tests for Classification and Prediction.* (Oxford University Press, 2003).

[27] Rao N, Brady SL, Chaudhuri G, Donzelli JJ, Wesling MW (2003). Gold-Standard? Analysis of the videofluoroscopic and fiberoptic endoscopic swallow examinations. J. Appl. Res..

[28] Umemneku Chikere CM, Wilson KJ, Allen AJ, Vale L (2021). Comparative diagnostic accuracy studies with an imperfect reference standard—A comparison of correction methods. BMC Med. Res. Methodol..

[29] Hui SL, Zhou XH (1998). Evaluation of diagnostic tests without gold standards. Stat. Methods Med. Res..

[30] Müller R, Büttner P (1994). A critical discussion of intraclass correlation coefficients. Statist. Med..

[31] Bland JM, Altman DG (1990). A note on the use of the intraclass correlation coefficient in the evaluation of agreement between two methods of measurement. Comput. Biol. Med..

[32] Rao NBSCG, Donzelli JJ, Wesling MW (2003). Gold-standard? Analysis of the videofluoroscopic and fiberoptic endoscopic swallow examinations. J. Appl. Res..

[33] Vacek PM (1985). The effect of conditional dependence on the evaluation of diagnostic tests. Biometrics.

[34] Staquet MRMLY, Muggia FM (1981). Methodology for the assessment of new dichotomous diagnostic tests. J. Chronic Dis..

[35] Buck AA, Gart JJ (1966). Comparison of a screening test and a reference test in epidemiologic studies: II. A probabilistic model for the comparison of diagnositc tests. Am. J. Epidemiol..

[36] Benner H (1996). Correcting for exposure misclassification using an alloyed gold standard. Epidemiology.

[37] Warrens MJ (2015). Five ways to look at Cohen's kappa. J. Psychol. Psychother..

[38] Vanbelle S, Albert A (2009). A note on the linearly weighted kappa coefficient for ordinal scales. Stat. Methodol..

[39] Williamson JM, Lipsitz SR, Manatunga AK (2000). Modeling kappa for measuring dependent categorical agreement data. (Oxford, England). Biostatistics.

[40] Linnet KBPMK, Reitsma JB (2012). Quantifying the accuracy of a diagnostic test or marker. Clin. Chem..

[41] Viera AJ (2005). Understanding interobserver agreement: The kappa statistic. Fam. Med..

[42] Dettori JR (2020). Detto Kappa and beyond: Is there agreement?. Glob. Spine J..

[43] Büttner P, Müller R (1994). A critical discussion of intraclass correlation coefficients. Stat. Med..

[44] Manor YGNCA, Fliss DM, Cohen JT (2007). Validation of a swallowing disturbance questionnaire for detecting dysphagia in patients with Parkinson's disease. Mov. Disord..

[45] Qu YTMKM (1996). Random effects models in latent class analysis for evaluating accuracy of diagnostic tests. Biometrics.

[46] Ahmad, W,. Awan, S., Asif, M. & Shakoor, A. Validation of EAT-10 questionnaire for assessment of dysphagia in patients with head and neck cancer. *Ayub Med. Coll. Abbottabad* 122–125 (2021).

[47] Ahn H K Y, Oh, J. E., Lee, J. H., & Lee, M. K.. Diagnsotic value of the eating assessment tool-10 for detecting dysphagia in head and neck cancer patients. *Annu. Rehabilit. Med.* 132–130 (2021).

[48] Conceição D F F, Cruz, P., Frade, I., Gramacho, J., Faias, S., & Claro, I. Fast track discharge after percutaneous endoscopic gastrostomy tube removal in head and neck cancer patients in oncological remission: a feasibility and safety study. *Endoscopy. *S343 (2023). 10.1016/j.gassur.2024.03.013 38555186

[49] Friedes CKJNNGJ, Burri R (2020). Late feeding tube dependency in head and neck cancer patients treated with definitive radiation therapy and concurrent systemic therapy. Cureus.

[50] Dechaphunkul TSPGSDAAJ (2022). Utility of prophylactic percutaneous endoscopic gastrostomy tube in head and neck cancer patients undergoing concurrent chemoradiation: A prospective observational cohort. Am. J. Otolaryngol..

