# Supplementary material for: The Arabic EAT-10 and FEES in dysphagia screening among cancer patients: a comparative prospective study
Source: Sci Rep. 2024 Apr 22;14:9258. doi: 10.1038/s41598-024-58572-z (PMC11035686; doi:10.1038/s41598-024-58572-z)
Supplement: Supplementary file 1 — Supplementary Information. [file 41598_2024_58572_MOESM1_ESM.docx]

**Supplementary File 1**

**Table S1:** data collection for A-EAT-10 and FEES during regular visits

| Treatment Plan | First Visit | Followup 1( after week from first visit) | Followup 2 ( after week from follow-up 1) | Follow-up 3  ( after week from follow-up 2) | Follow-up 4  ( after week from follow-up 3) | Follow-up 5  (after week from follow-up 4) | Follow-up 6 (after month) |
| --- | --- | --- | --- | --- | --- | --- | --- |
| **Radiation** | General swallowing assessment  (A-EAT-10)  And FEES | Regular follow-up | Currently, the pt. end 7^th^ sessions of radiation (apply A-EAT-10) | Currently, the pt. end 15-16^th^ sessions of radiation  (apply A-EAT-10) | Currently, the pt. end 22th-23 sessions of radiation  (apply A-EAT-10) | At the end of radiation (30 or 33 sessions)  (apply A-EAT-10) | Month after last session of Tx  (apply A-EAT-10 and FEES |
| **Radical concurrent chemoradiotherapy** | General swallowing assessment  (A-EAT-10)  And FEES | Regular follow-up | at the initial visit, the patient had completed the 7th sessions of radiation, and he had undergone first cycle of chemotherapy. Then apply A-eat-10 | at the second visit, the patient had completed the 14^th^-15 th sessions of radiation, and he had undergone second cycle of chemotherapy. Then apply A-eat-10 | at the 3rd visit, the patient had completed the 22th-23 sessions of radiation, and he had undergone third cycle of chemotherapy. Then apply A-eat-10 | at this stage, the patient has finsihed cycles of chemotherapy and underwent the finals sessions of radiotherapy (30^th^ to 33 rd) we apply A-EAT-10 | Month after finishing last radio sessions  (apply A-EAT-10 and FEES |
| **Surgery** | General swallowing assessment  (A-EAT-10)  And FEES | Regular follow-up | When physician allow to start oral feeding apply A-EAT-10) | Depending on the patient's progress, oral feeding typically begins with a one-week water intake period. During this stage, we administer the A-EAT-10 assessment once. After receiving approval from the physician to introduce a soft diet, we conduct another A-EAT-10 assessment a few days after the patient starts the soft diet. The final assessment is conducted after one month | | | After 1-months from date of soft diet intake.  apply A-EAT-10 and FEES |

The mathematical and statistical analysis protocol:

*Kappa Coefficient*:

There is no standard way to assess the strength of the agreement. However, several studies suggested the following scale[65, 66]:

**Table S2**: Common interpretation of kappa values

| Kappa | Agreement |
| --- | --- |
| ≤ 0.2 | Poor |
| 0.21–0.4 | Fair |
| 0.41–0.6 | Moderate |
| 0.61–0.8 | Good |
| 0.81–0.99 | Almost perfect |
| 1 | Perfect |

Imperfect gold standard correction

Consider **Table S3**, which shows a classical 2×2 contingency table for a new test vs the gold standard. Under the gold standard perfection assumption, the sensitivity-specificity test will be straightforward (equations 1–3). As explained before, this is not the case for FEES.

Table S3: Methods of diagnostic agreement cross-tabulation and marginal totals

|  | | FEES* test results (gold standard) | |  |
| --- | --- | --- | --- | --- |
|  |  | Dysphagia = 1 | Normal = 0 | Total |
| EAT-10^ test results (new test) | Dysphagia = 1 | A | b | a + b = g |
|  | Normal = 0 | C | d | c + d = h |
|  | Total | a + c = e | b + d = f | a + b + c + d = N |

*FEES: Fibre-optic Evaluation of Swallowing

^EAT-10: Arabic EAT-10

The correction calculations are described in the following paragraphs, with the notations explained in Tables S3.

**Table S4**: Table of notations

| Notation | Meaning |
| --- | --- |
| ${Sn}_{F}$ | Sensitivity of FEES* test |
| ${Sn}_{E}$ | Sensitivity of EAT-10** test |
| ${Sp}_{F}$ | Specificity of FEES test |
| ${Sp}_{E}$ | Specificity of A-EAT-10 test |
| ${Sn}_{cE}$ | Corrected Sensitivity of A-EAT-10 |
| ${Sp}_{cE}$ | Corrected Specificity of A-EAT-10 |
| $\hat{P}$ | The estimated prevalence of dysphagia in the population |
| $P_{rs}$ | Prevalence of dysphagia in the sample |

*FEES: Fiber-optic Evaluation of Swallowing

**EAT-10: Arabic Eat-10

$${Sn}_{E}=\frac{a}{e} \ldots.. (1)$$

$${Sp}_{E}=\frac{d}{f}\ldots.. (2)$$

$$P_{rs}=\frac{e}{N}\ldots.. (3)$$

Correction equation based on Staquet et al.[35] As follows:

$${Sn}_{cE}=\frac{g{Sp}_{F}-b}{N\left( {Sp}_{F}-1 \right)+e}\ldots.. (4)$$

$${Sp}_{cE}=\frac{h{Sn}_{F}-c}{N{Sn}_{F}-e}\ldots.. (5)$$

$$\hat{P}=\frac{N\left( {Sp}_{F}-1 \right)+e}{N({Sn}_{F}+{Sp}_{F}-1)}\ldots.. (6)$$
